# Epidemiological characteristics and genetic evolution of rhinovirus in Jining City, 2024–2025

**DOI:** 10.3389/fmicb.2026.1927704

**Published:** 2026-09-04

**Authors:** Mingsheng Zhao, Tongyun Song, Wen Li, Yajuan Jiang, Yongjian Jia, Huixin Dou, Ying Yue, Boyan Jiao, Xiaoyu Wang

**Affiliations:** 1 Institute of Immunology and Molecular Medicine, Jining Medical University, Jining, China; 2 Department of Laboratory, Rencheng Maternal and Child Health Hospital, Jining, China; 3 College of Clinical and Basic Medicine, Shandong First Medical University and Shandong Academy of Medical Sciences, Jinan, China; 4 Department of Microbiological Laboratory, Jining Center for Disease Control and Prevention, Jining, China; 5 Department of Infectious Disease Control, Jining Center for Disease Control and Prevention, Jining, China

**Keywords:** genetic evolution, molecular characterization, molecular epidemiology, respiratory virus, rhinovirus

## Abstract

**Objective:**

To investigate the epidemiological characteristics and genetic evolution of rhinovirus (RV) among patients with influenza-like illness (ILI) in Jining City, Shandong Province, from 2024 to 2025, and to provide scientific evidence for the formulation of regional respiratory virus prevention and control strategies.

**Methods:**

A total of 2,724 throat swab specimens were collected from ILI cases at Jining First People’s Hospital between 2024 and 2025. Ten respiratory viruses were screened using real-time reverse transcription-quantitative PCR. Of 101 RV-positive specimens, 30 with lower cycle threshold (*Ct*) values were subjected to whole-genome targeted enrichment sequencing. Molecular typing was performed based on VP1 region nucleotide sequences. A maximum likelihood (ML) phylogenetic tree was constructed, and genetic distances and key amino acid substitution sites were analyzed.

**Results:**

Among the 2,724 specimens, influenza virus had the highest detection rate (14.98%), followed by SARS-CoV-2 (7.05%), with rhinovirus ranking third (3.71%, 101/2,724). The RV-positive rate in 2025 (5.53%) was significantly higher than in 2024 (1.95%; χ^2^ = 23.55, *P* < 0.001). An unusually high RV positivity rate (23.53%) was observed in November 2025. The 30 sequenced strains were classified into RV-A (73.33%, 17 genotypes), RV-B (10.00%, 3 genotypes), and RV-C (16.67%, 3 genotypes), with RV-A as the predominant species and genotypes exhibiting high diversity. Phylogenetic analysis revealed co-circulation of multiple species and genotypes, with 2025 strains distributed across a broader range of the phylogenetic tree. The nucleotide similarity between the VP1 regions of the 30 strains and their respective reference strains ranged from 86.08% to 95.37%, and amino acid similarity from 91.58% to 99.65%, with 1–24 amino acid substitution sites per strain.

**Conclusion:**

RV-A was the predominant rhinovirus species circulating in Jining City during 2024–2025, with high genotypic diversity. The detection rate in 2025 increased significantly compared with 2024, accompanied by an autumn epidemic peak. Rhinoviruses in this region exhibit ongoing evolution in the VP1 region. Enhanced molecular surveillance is warranted to dynamically monitor epidemiological and evolutionary trends.

## 1 Introduction

Rhinovirus (RV), belonging to the genus Enterovirus within the family Picornaviridae, is one of the most common pathogens causing acute upper respiratory tract infections in humans. RV is a non-enveloped, single-stranded positive-sense RNA virus with a genome length of approximately 7.1–7.2 kb, containing a single large open reading frame that encodes four structural proteins (VP1–VP4) and seven non-structural proteins. Based on genetic and antigenic characteristics, rhinoviruses are classified into three species: RV-A, RV-B, and RV-C. RV-A comprises at least 80 genotypes, RV-B at least 32 genotypes, and RV-C, first identified in 2006, at least 57 genotypes. All three species co-circulate globally (McIntyre et al., 2013; Palmenberg et al., 2009; Zell et al., 2017).

Rhinovirus infection is exceedingly common worldwide. Virtually every child experiences at least one RV infection by the age of 2 years, and adults are infected an average of two to three times per year. Although the majority of RV infections manifest as self-limiting common cold symptoms, in infants, the elderly, and individuals with chronic respiratory diseases, RV infection can cause severe lower respiratory tract illnesses, including bronchiolitis, pneumonia, and acute exacerbations of asthma (Gern, 2010; Jacobs et al., 2013). Recent studies have further demonstrated that rhinoviruses, particularly RV-C, are closely associated with recurrent wheezing and the development and progression of childhood asthma, and are considered one of the most important viral triggers of acute asthma exacerbations in children (Bisgaard et al., 2010). In addition, RV infection is closely linked to acute exacerbations of chronic obstructive pulmonary disease (COPD), sinusitis, and otitis media, imposing a substantial burden on global public health (Mallia et al., 2011).

Rhinovirus can be detected throughout the year, but its epidemic seasonality varies by geographic region and climate. In temperate regions, RV infections typically peak in spring and autumn, whereas in tropical and subtropical regions, the rainy season predominates (Morikawa et al., 2015). In China, RV is among the viruses with the highest detection rates in ILI and acute respiratory infection (ARI) cases. However, substantial inter-regional and inter-annual variation exists in epidemic intensity and species distribution, and long-term, systematic molecular epidemiological surveillance data remain limited (Li et al., 2021). Notably, in the post-COVID-19 era, the implementation and subsequent relaxation of non-pharmaceutical interventions (NPIs) have profoundly impacted the epidemiological landscape of respiratory viruses. An “immunity gap” resulting from prolonged NPI implementation has led to off-season or unusually high epidemics of certain respiratory viruses, and rhinovirus has likewise exhibited epidemiological patterns distinct from those observed previously (Olsen et al., 2021).

Jining City, located in southwestern Shandong Province, lies within a warm-temperate monsoon climate zone. With a dense population, the region faces considerable pressure in the prevention and control of respiratory infectious diseases. Jining First People’s Hospital serves as a national-level ILI sentinel surveillance hospital, and the Jining Center for Disease Control and Prevention functions as a national-level influenza surveillance network laboratory, both of which have long undertaken respiratory virus surveillance in the region. However, molecular epidemiological studies of rhinovirus in Jining remain lacking, and systematic understanding of species distribution, genetic diversity, and evolutionary trends is absent. To address this gap, the present study utilized ILI surveillance data from Jining City for 2024–2025 to delineate the epidemiological characteristics of rhinovirus, performed whole-genome sequencing of representative strains, and systematically analyzed molecular typing, phylogenetic relationships, and amino acid substitution patterns. The findings aim to provide scientific evidence for the formulation of regional respiratory virus prevention and control strategies and for vaccine development.

## 2 Materials and methods

### 2.1 Specimen collection

Throat swab specimens were collected from ILI cases at Jining First People’s Hospital (a national-level ILI sentinel surveillance hospital) between 2024 and 2025. After collection, specimens were transported under cold-chain conditions (2–8 °C) within two working days to the Jining Center for Disease Control and Prevention (a national-level influenza surveillance network laboratory) for respiratory multi-pathogen nucleic acid testing. Specimens that could not be delivered to the laboratory within 48 h were stored at −70 °C or below and delivered within 1 week.

### 2.2 Nucleic acid testing

Nucleic acids were extracted from throat swab specimens using the BioPerfectus automated nucleic acid extraction system. Ten respiratory viruses – rhinovirus (RV), SARS-CoV-2, influenza virus (Flu), respiratory syncytial virus (RSV), human adenovirus (HAdV), human metapneumovirus (HMPV), human parainfluenza virus (HPIV), human common coronavirus (HCoV), human bocavirus (HBoV), and enterovirus (EV) – were detected using a respiratory multi-pathogen nucleic acid detection kit (BioGerm) by real-time fluorescence quantitative PCR.

### 2.3 Rhinovirus whole-genome sequencing

Thirty RV-positive specimens with lower *Ct* values were selected for whole-genome sequencing (Prioritizing specimens with low *Ct* values for sequencing was a methodological necessity rather than an intentional sampling bias. Because the sequencing success rate of high *Ct* samples drops significantly, low *Ct* specimens were chosen to ensure adequate template quality, thereby facilitating successful amplification and sequencing). Library construction and targeted enrichment were performed using a rhinovirus whole-genome enrichment library preparation kit (HONGWEITES). Briefly, extracted RV RNA (10 ng–1 μg) was fragmented at 94 °C for 7 min, followed by first-strand cDNA synthesis (25 °C for 10 min, 42 °C for 15 min, 70 °C for 15 min), second-strand synthesis, and 3’-end A-tailing (16 °C for 30 min, 72 °C for 15 min). After ligation of Illumina-specific adapters (20 °C for 15 min), the products were purified using 0.45 × VAHTS DNA Clean Beads (Vazyme). The ligation products underwent Pre-PCR amplification (98 °C for 1 min; 98 °C for 10 s, 60 °C for 30 s, 72°C for 30 s, 7–12 cycles; 72 °C for 5 min) and were purified with 0.9× magnetic beads. Library aliquots of 750 ng (single library) or 500 ng per library (pooled hybridization) were mixed with biotin-labeled rhinovirus whole-genome probes, denatured at 85 °C for 5 min, and hybridized at 60 °C for 12–18 h. Hybridization products were captured using Capture Beads provided in the kit, washed five times with pre-warmed (60 °C) Wash Buffer, and subjected to Post-PCR amplification (95 °C for 1 min; 98°C for 20 s, 60°C for 30 s, 72 °C for 30 s, 19–22 cycles; 72 °C for 5 min). The final products were purified with 1.1× magnetic beads. Whole-genome sequencing was performed on an Illumina NextSeq 2000 platform using the P1 Reagent Kit (300 cycles). The sequencing coverage for all 30 specimens was ≥99%, with an average depth ranging from 55X to 62409X (median 6871X), and the Q30 proportion ranged from 86.45% to 96.90% (mean 91.5%). These results indicate that the sequencing data quality is high and fully satisfies the requirements for subsequent bioinformatics analyses.

### 2.4 Sequence assembly and bioinformatics analysis

Raw sequencing data were assembled and analyzed using CLC Genomics Workbench 24. Reference sequences for each rhinovirus species were downloaded from the NCBI database. Sequence alignment was performed using MEGA 11 and BioEdit software. Phylogenetic trees were constructed using the Maximum Likelihood (ML) method implemented in IQ-TREE (v3.1.3). The best-fit nucleotide substitution model was automatically selected via the ModelFinder module based on the Bayesian Information Criterion (BIC), and the GTR+F+R8 model was ultimately chosen. Node branch supports were evaluated using 1,000 ultrafast bootstrap (UFBoot) replicates and 1,000 Shimodaira-Hasegawa approximate likelihood ratio test (SH-aLRT) replicates. Missing data were treated with partial deletion. Genetic distances and similarities among the three genotypes (RV-A, RV-B, and RV-C) were analyzed using MEGA 11. Nucleotide similarities between each strain and its corresponding genotype reference strain were determined. Intra-genotype genetic distances were calculated under the p-distance model with uniform rates among sites; gaps and missing data were treated by partial deletion, retaining sites with ≥95% coverage. Key amino acid substitution sites were analyzed using MEGA 11 and BioEdit software. To screen for recombination events across the whole-genome sequences of the RV-A, RV-B, and RV-C groups (comprising both study samples and reference sequences), RDP4 software was employed. Detection was performed using six algorithms built into the software: RDP, GENECONV, BOOTSCAN, MaxChi, Chimaera, and SisScan. To enhance the reliability of the findings, a recombination event was considered positive only when it was simultaneously supported by at least three algorithms (*P* < 0.05, with Bonferroni correction), and all detected events were manually verified on a case-by-case basis.

### 2.5 Statistical analysis

Statistical analyses were conducted with SPSS, version 24.0. Between-group differences were assessed using the χ^2^ test for categorical variables and one-way ANOVA for continuous variables. Significance was defined as *P* < 0.05.

## 3 Results

### 3.1 Detection of respiratory viruses

Between 2024 and 2025, a total of 2,724 ILI cases were enrolled in this study, comprising 1,387 cases in 2024 and 1,337 cases in 2025. The detection results for the ten respiratory viruses are shown in Table 1. Influenza virus (Flu) had the highest detection rate, with 408 positive cases (14.98%), followed by SARS-CoV-2 with 192 cases (7.05%), and rhinovirus (RV) ranking third with 101 cases (3.71%). The detection rates of other respiratory viruses, in descending order, were: respiratory syncytial virus (RSV) 37 cases (1.36%), human common coronavirus (HCoV) 33 cases (1.21%), human parainfluenza virus (HPIV) 24 cases (0.88%), human metapneumovirus (HMPV) 22 cases (0.81%), human adenovirus (HAdV) 16 cases (0.59%), and enterovirus (EV) 6 cases (0.22%). Human bocavirus (HBoV) was not detected.

**TABLE 1:** Detection of respiratory viruses in ILI cases in Jining City, 2024–2025.

Year	Total cases	SARS-CoV-2		Flu		RSV		HAdV		HMPV		HPIV		HCoV		HBoV		RV		EV	
		**Pn**	**Pr**	**Pn**	**Pr**	**Pn**	**Pr**	**Pn**	**Pr**	**Pn**	**Pr**	**Pn**	**Pr**	**Pn**	**Pr**	**Pn**	**Pr**	**Pn**	**Pr**	**Pn**	**Pr**
2024	1387	113	8.15%	230	16.58%	9	0.65%	14	1.01%	16	1.15%	16	1.15%	4	0.29%	0	0%	27	1.95%	4	0.29%
2025	1337	79	5.91%	178	13.31%	28	2.09%	2	0.15%	6	0.45%	8	0.60%	29	2.17%	0	0%	74	5.53%	2	0.15%
Total	2724	192	7.05%	408	14.98%	37	1.36%	16	0.59%	22	0.81%	24	0.88%	33	1.21%	0	0%	101	3.71%	6	0.22%

Pn, number of positive cases; Pr, positive rate. SARS-CoV-2, severe acute respiratory syndrome coronavirus 2; Flu, influenza virus; RSV, respiratory syncytial virus; HAdV, human adenovirus; HMPV, human metapneumovirus; HPIV, human parainfluenza virus; HCoV, human coronavirus; HBoV, human bocavirus; RV, rhinovirus; EV, enterovirus.

In 2024, 27 RV-positive cases were detected, yielding a positivity rate of 1.95%; in 2025, 74 cases were detected, with a positivity rate of 5.53%. The RV detection rate in 2025 was significantly higher than that in 2024 (χ^2^ = 23.55, *P* < 0.001). The detection rates of RSV (28 cases, 2.09%) and HCoV (29 cases, 2.17%) also increased in 2025 compared with 2024 (χ^2^ = 9.56, *P* < 0.05; χ^2^ = 18.58, *P* < 0.001, respectively), whereas the detection rates of SARS-CoV-2 and Flu showed a declining trend (χ^2^ = 4.87, *P* < 0.05; χ^2^ = 5.46, *P* < 0.05, respectively).

### 3.2 Epidemiological characteristics of rhinovirus

The RV detection rate in 2025 was significantly higher than in 2024 (χ^2^ = 23.55, *P* < 0.001) (Table 1). Regarding seasonal distribution (Supplementary Table 1), RV was detected in all four seasons in 2024: 8 cases in spring (March–May, positivity rate 2.05%), 4 cases in summer (June–August, 3.05%), 4 cases in autumn (September–November, 1.00%), and 11 cases in winter (December–February, 2.36%), with no statistically significant difference among seasons (χ^2^ = 3.14, *P* = 0.371). In 2025, the seasonal distribution differed significantly (χ^2^ = 35.64, *P* < 0.001): autumn had the highest detection rate with 43 cases (positivity rate 11.38%), followed by winter with 13 cases (2.58%), spring with 10 cases (3.44%), and summer with 8 cases (4.85%). The RV detection rate in autumn 2025 was significantly higher than that in autumn 2024 (χ^2^ = 34.95, *P* < 0.001); no statistically significant differences were observed between the 2 years for the other seasons.

**FIGURE 1:**
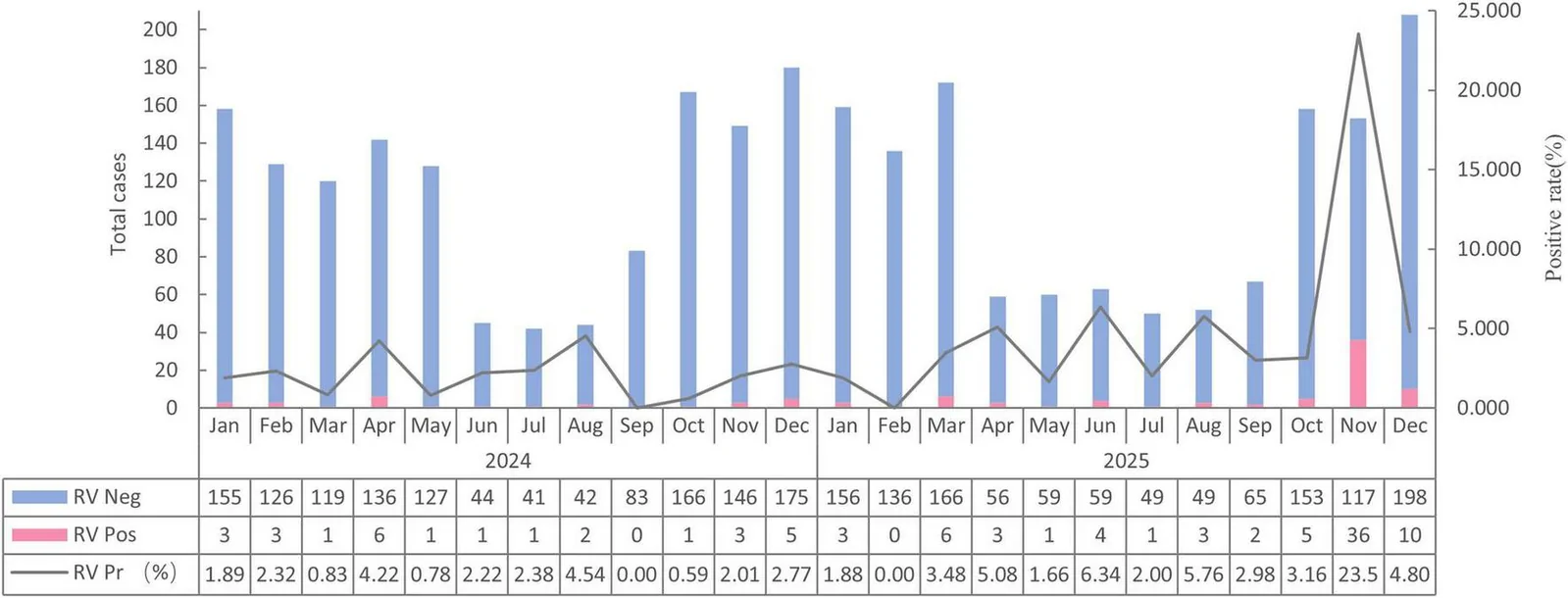
Temporal distribution of rhinovirus detection among ILI cases in Jining City, 2024–2025. RV Pos, rhinovirus-positive cases; RV Neg, rhinovirus-negative cases; RV Pr, rhinovirus positivity rate.

Monthly distribution analysis (Figure 1 and Supplementary Table 1) showed that RV was detected sporadically throughout 2024, with 0–6 cases per month and no apparent epidemic peak. In 2025, an unusually high number of RV cases was observed in November, with 36 cases detected in a single month, yielding a positivity rate of 23.53% and forming a distinct epidemic peak; the remaining months had 0–10 cases each, reflecting a sporadic pattern.

With regard to sex distribution (Supplementary Table 1), in 2024, among 625 male ILI cases, 16 were RV-positive (2.56%), and among 762 female ILI cases, 11 were RV-positive (1.44%), with no statistically significant difference between sexes (χ^2^ = 1.70, *P* = 0.193). In 2025, among 630 male cases, 37 were RV-positive (5.87%), and among 707 female cases, 37 were RV-positive (5.23%), again with no statistically significant sex difference (χ^2^ = 0.15, *P* = 0.696).

### 3.3 Molecular typing of rhinovirus

Thirty RV-positive specimens with lower Ct values were selected for whole-genome sequencing, and molecular typing was performed based on VP1 region nucleotide sequences (The VP1 region exhibits the highest variability among rhinovirus capsid proteins, containing major neutralizing antigenic sites and receptor-binding domains. Its sequence information provides sufficient resolution to accurately distinguish different rhinovirus types, and it has been universally recognized as the standard target for rhinovirus genotyping) (Laine et al., 2006; Ledford et al., 2004; McIntyre et al., 2013) (Table 2). The results showed that the 30 rhinovirus strains belonged to three species comprising 23 genotypes. Specifically, 22 strains (73.33%) were RV-A, encompassing 17 genotypes, with A40 (3 strains) and A1, A30, and A31 (2 strains each) were predominant; the remaining genotypes (A2, A8, A10, A12, A13, A18, A25, A43, A49, A61, A80, A100, A105) were each represented by a single strain. Three strains (10.00%) were RV-B, belonging to genotypes B4, B14, and B91 (one strain each). Five strains (16.67%) were RV-C, belonging to genotypes C3 (3 strains), C13 (1 strain), and C43 (1 strain). RV-A was the predominant species in this region, with genotypes exhibiting high diversity.

**TABLE 2:** Rhinovirus typing based on the VP1 region.

Type	Number of samples	Sample ID	Type
A	22	Shandong/Jining/1143/2024	A8
B	3	Shandong/Jining/1311/2024	A30
C	5	Shandong/Jining/1376/2024	A18
A1	2	Shandong/Jining/1410/2024	A25
A2	1	Shandong/Jining/1895/2024	C43
A8	1	Shandong/Jining/2006/2024	A100
A10	1	Shandong/Jining/2256/2024	A12
A12	1	Shandong/Jining/2310/2024	A31
A13	1	Shandong/Jining/2362/2024	A31
A18	1	Shandong/Jining/2416/2024	C13
A25	1	Shandong/Jining/108/2025	B4
A30	2	Shandong/Jining/110/2025	B14
A31	2	Shandong/Jining/124/2025	A80
A40	3	Shandong/Jining/2435/2024	A61
A43	1	Shandong/Jining/543/2025	A30
A49	1	Shandong/Jining/546/2025	A1
A61	1	Shandong/Jining/933/2025	A10
A80	1	Shandong/Jining/1056/2025	A40
A100	1	Shandong/Jining/1090/2025	A1
A105	1	Shandong/Jining/1544/2025	A40
B4	1	Shandong/Jining/1547/2025	B91
B14	1	Shandong/Jining/1550/2025	C3
B91	1	Shandong/Jining/1557/2025	A40
C3	3	Shandong/Jining/1617/2025	C3
C13	1	Shandong/Jining/1619/2025	A13
C43	1	Shandong/Jining/1621/2025	C3
		Shandong/Jining/1630/2025	A49
		Shandong/Jining/1694/2025	A2
		Shandong/Jining/1697/2025	A105
		Shandong/Jining/2048/2025	A43

### 3.4 Phylogenetic analysis

#### 3.4.1 Phylogenetic analysis based on whole-genome sequences

**FIGURE 2:**
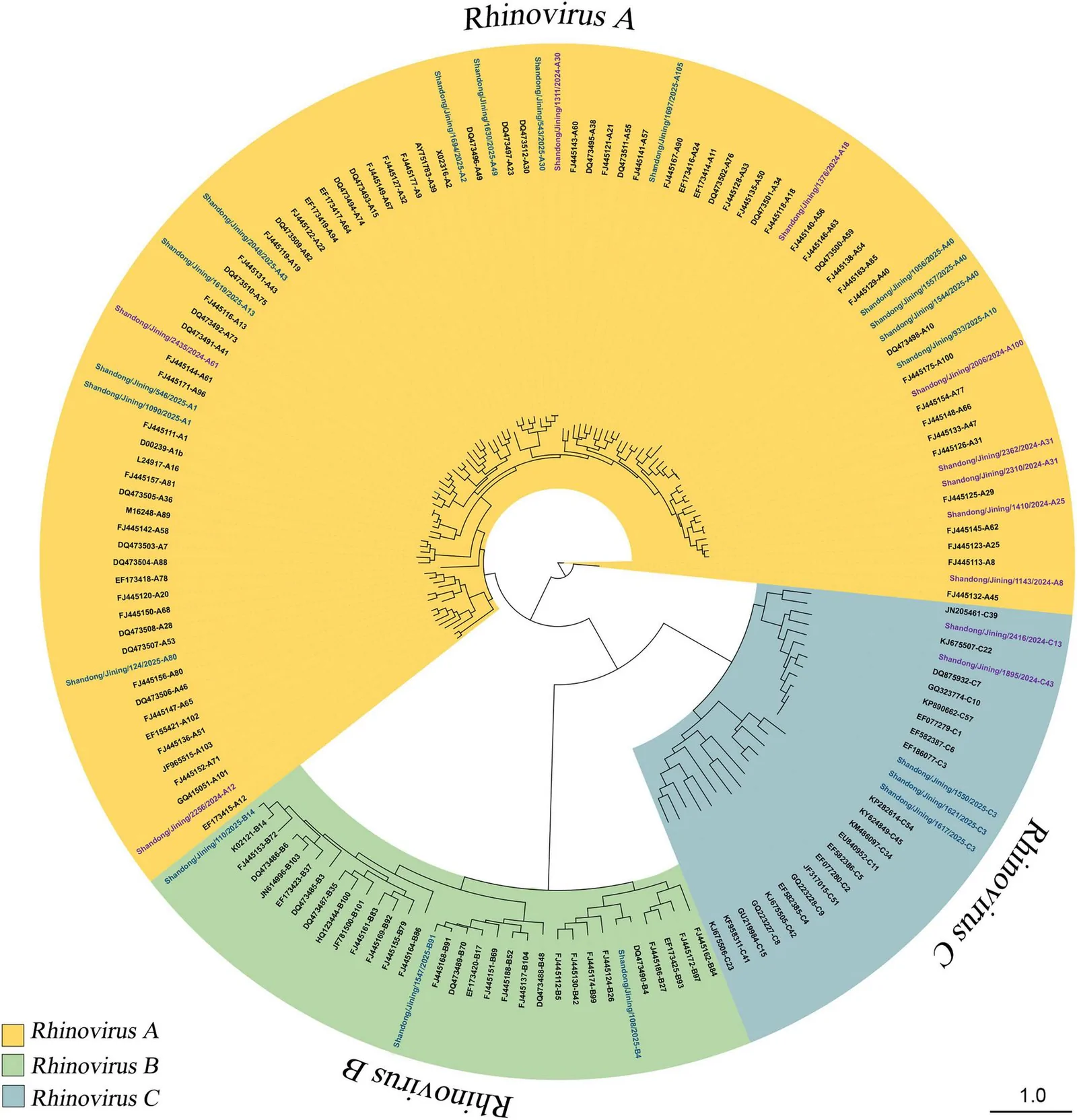
Phylogenetic tree based on rhinovirus whole-genome sequences. Maximum likelihood phylogenetic trees constructed using IQ-TREE (v3.1.3) under the GTR+F+R8 model. Branch supports were assessed using 1,000 ultrafast bootstrap replicates and 1,000 SH-aLRT replicates. Values at the nodes represent bootstrap supports / SH-aLRT support values. Yellow-shaded area: HRV-A strains; green-shaded area: HRV-B strains; blue-shaded area: HRV-C strains. Purple labels: rhinovirus strains sequenced in 2024; blue labels: rhinovirus strains sequenced in 2025; black labels: rhinovirus reference strains downloaded from NCBI.

To investigate the genetic evolutionary relationships among the rhinovirus sequences obtained in this study, an ML phylogenetic tree was constructed based on whole-genome sequences (Figure 2). The results showed that all rhinovirus strains clustered clearly into three distinct branches, corresponding to HRV-A, HRV-B, and HRV-C, demonstrating that the three species are genetically divergent at the whole-genome level. In the phylogenetic tree, the HRV-A clade (yellow-shaded area in Figure 2) contained the largest number of strains. The 22 HRV-A strains obtained in this study (2024 strains labeled in purple, 2025 strains labeled in blue) clustered closely with HRV-A reference sequences downloaded from the NCBI database, forming multiple subclades. The HRV-A strains from this study were distributed across different subclades, indicating high genetic diversity among HRV-A strains in this region and suggesting the potential co-circulation of multiple distinct transmission chains during the same period. The HRV-B clade (green-shaded area in Figure 2) contained relatively few strains. The three HRV-B strains from this study clustered within the same branch as the reference strains, exhibiting relatively conserved genetic characteristics. The HRV-B clade was clearly separated from the HRV-A and HRV-C clades, further confirming the substantial genetic distance among different rhinovirus species. The HRV-C clade (blue-shaded area in Figure 2) grouped the HRV-C strains identified in this study; these strains formed independent evolutionary branches with NCBI reference strains. In this study, the 2024 and 2025 HRV-C strains were distributed at different positions within the phylogenetic tree, suggesting the persistent circulation of HRV-C in this region with a potential degree of genetic variation. From a temporal perspective, strains from both 2024 and 2025 were distributed across different clades of the phylogenetic tree, indicating that all three rhinovirus species circulated during both surveillance years. The phylogenetic analysis demonstrates that the rhinoviruses circulating in this region possess rich genetic diversity, with strains of all three species co-circulating and multiple genotypes co-circulating within the same species. Whole-genome phylogenetic analysis provides an important basis for a deeper understanding of the molecular epidemiological characteristics and genetic evolutionary patterns of rhinovirus in this region.

Recombination analysis of the whole-genome sequences of the study strains and reference strains across various subtypes identified 10 reliable recombination events across the RV-A, RV-B, and RV-C analytical groups (supported by three or more algorithms, *P* < 0.05). These recombination events involved multiple serotypes and genotypes from RV-A (A8, A18, A25, A31, A40, A80, A100, and A105) and RV-B (B4 and B14). These findings indicate active genetic recombination of rhinoviruses during their circulation in the Jining region, which likely serves as a crucial mechanism driving their genetic diversity. Recombination hotspots were predominantly localized within the genomic regions encoding viral capsid proteins (VP4, VP2, and VP1). Such recombination events may alter viral antigenic properties and pathogenicity, thereby posing significant implications for vaccine development and disease prevention and control (Supplementary Table 2).

#### 3.4.2 Phylogenetic analysis based on VP1 region sequences

**FIGURE 3:**
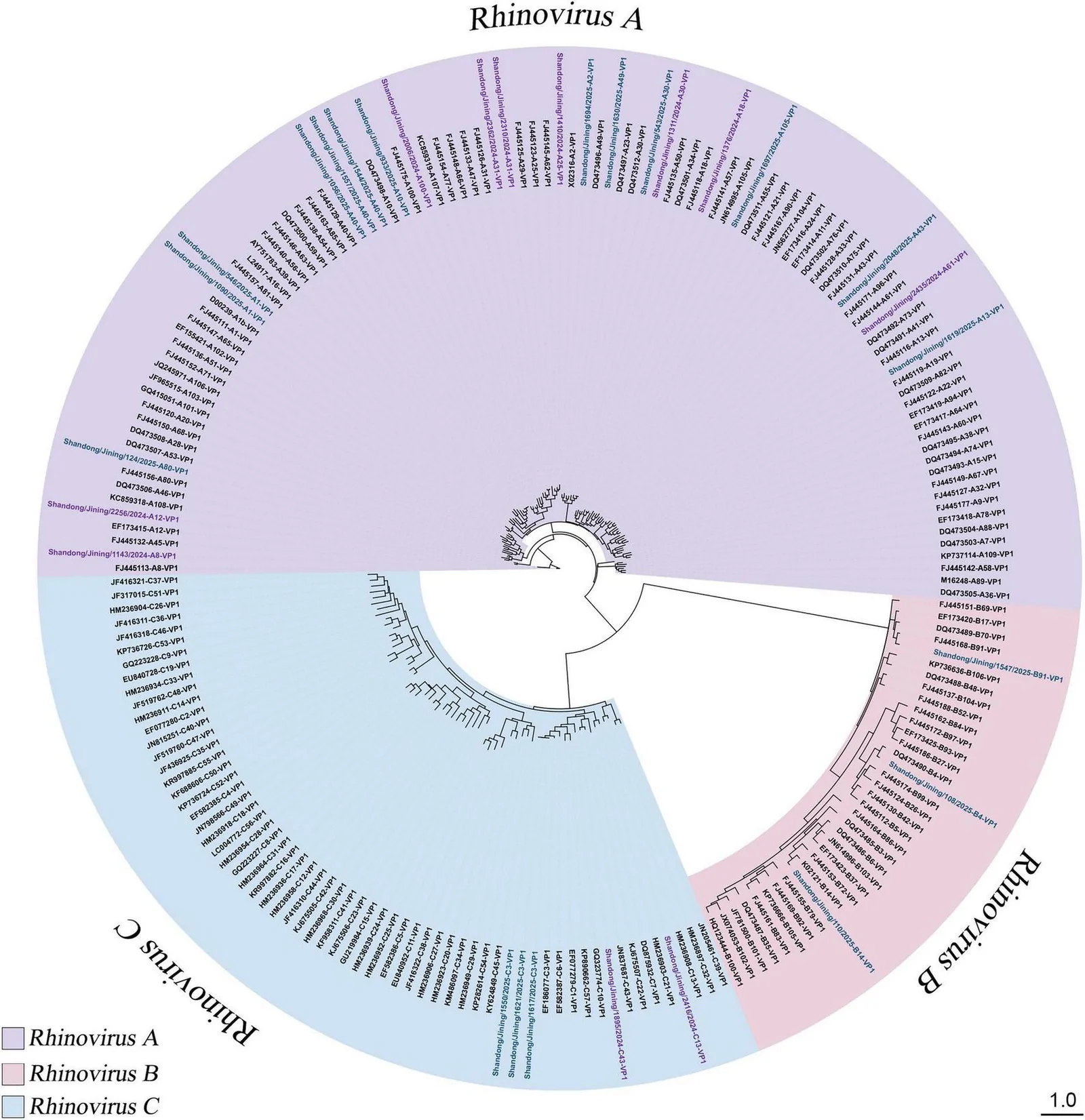
Phylogenetic tree based on rhinovirus VP1 region sequences. Maximum likelihood phylogenetic trees constructed using IQ-TREE (v3.1.3) under the GTR+F+R8 model. Branch supports were assessed using 1,000 ultrafast bootstrap replicates and 1,000 SH-aLRT replicates. Values at the nodes represent bootstrap supports / SH-aLRT support values. Purple-shaded area: HRV-A strains; pink-shaded area: HRV-B strains; blue-shaded area: HRV-C strains. Purple labels: rhinovirus strains sequenced in 2024; blue labels: rhinovirus strains sequenced in 2025; black labels: rhinovirus reference strains downloaded from NCBI.

Phylogenetic analysis based on the VP1 region (Figure 3) showed that rhinoviruses were clearly divided into three major clades corresponding to HRV-A, HRV-B, and HRV-C. In the tree, HRV-A strains (purple-shaded area) formed a large cluster; HRV-B strains (pink-shaded area) constituted an independent evolutionary branch; and HRV-C strains (blue-shaded area) formed the third major clade. The 2024 strains (purple labels) and 2025 strains (blue labels) exhibited marked genetic diversity in their phylogenetic distribution. The 2024 strains were predominantly clustered within the HRV-A clade and showed high sequence homology with reference strains downloaded from NCBI (black labels), indicating close genetic relatedness to known circulating strains. The phylogenetic analysis further indicated that the newly isolated 2025 strains were distributed across a broader range of the tree, with some strains forming independent evolutionary subclades distinct from known reference strains, suggesting that these strains may harbor unique genetic variation characteristics. Notably, a certain genetic distance existed among different strains isolated during the same period, reflecting the continuous evolution and diversification of rhinoviruses in nature. The differences in the phylogenetic positions of strains isolated in different years reveal the dynamic changes in the genetic structure of the rhinovirus population, providing important molecular epidemiological data for monitoring viral evolutionary trends.

### 3.5 Genetic distance and similarity analysis

**FIGURE 4:**
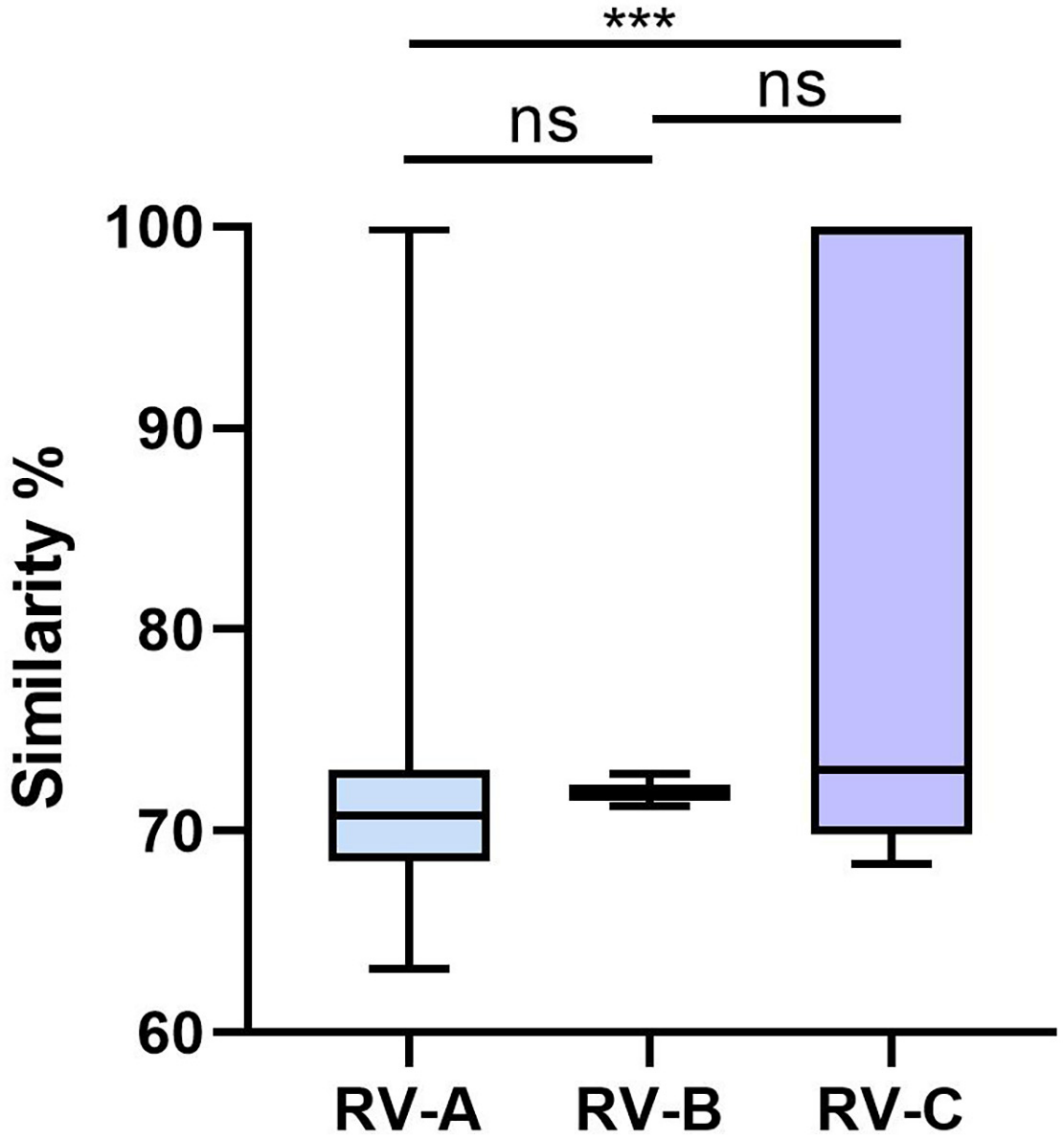
VP1 region sequence similarity analysis of rhinovirus species A, B, and C in Jining City, 2024–2025. Data are shown as mean ± SEM. One-way ANOVA with Bonferroni correction was used for intergroup comparisons. ***P < 0.001; ns, not significant.

Among the 30 rhinovirus strains sequenced in this study, the mean genetic distance of VP1 region sequences was 0.29 for the 22 RV-A strains, 0.28 for the 3 RV-B strains, and 0.20 for the 5 RV-C strains. Intra-species similarity analysis for RV-A, RV-B, and RV-C is presented in Figure 4.

Similarity analysis comparing the VP1 region sequences of the 30 study strains with their respective species-specific reference strains (Table 3) showed that the nucleotide similarity ranged from 86.08% to 95.37% (mean, 89.93%), and the amino acid similarity ranged from 91.58% to 99.65% (mean, 96.13%). For the 22 RV-A strains, nucleotide similarity with their respective reference strains ranged from 86.08% to 95.37% (mean, 89.76%), and amino acid similarity from 91.58% to 99.65% (mean, 96.10%). For the 3 RV-B strains, nucleotide similarity ranged from 87.77% to 89.30% (mean, 88.76%), and amino acid similarity from 93.43% to 96.62% (mean, 95.19%). For the 5 RV-C strains, nucleotide similarity ranged from 88.73% to 92.61% (mean, 91.40%), and amino acid similarity from 95.20% to 98.53% (mean, 96.78%). Amino acid similarity was consistently higher than nucleotide similarity for all strains, indicating that the majority of nucleotide substitutions in the VP1 region are synonymous mutations. Notably, strain Shandong/Jining/1697/2025 (genotype A105) exhibited the highest nucleotide similarity (95.37%) and amino acid similarity (99.65%) with reference strain JN614995, with only one amino acid substitution (N90D), suggesting an extremely close genetic relationship between this strain and the reference strain.

**TABLE 3:** Similarity analysis of VP1 region sequences between study strains and reference strains in Jining City, 2024–2025.

Sample ID	Reference sequence	Type	Nucleotide identity (%)	Protein identity (%)
Shandong/Jining/1143/2024	FJ445113	A8	89.79	93.66
Shandong/Jining/1311/2024	DQ473512	A30	89.75	97.53
Shandong/Jining/1376/2024	FJ445118	A18	90.48	97.91
Shandong/Jining/1410/2024	FJ445123	A25	86.88	92.20
Shandong/Jining/1895/2024	JN837687	C43	90.44	98.53
Shandong/Jining/2006/2024	FJ445175	A100	91.78	98.61
Shandong/Jining/2256/2024	EF173415	A12	89.43	98.26
Shandong/Jining/2310/2024	FJ445126	A31	86.08	91.58
Shandong/Jining/2362/2024	FJ445126	A31	86.43	91.58
Shandong/Jining/2416/2024	HM236908	C13	88.73	95.20
Shandong/Jining/108/2025	DQ473490	B4	89.20	95.52
Shandong/Jining/110/2025	K02121	B14	87.77	93.43
Shandong/Jining/124/2025	FJ445156	A80	91.21	97.95
Shandong/Jining/2435/2024	FJ445144	A61	90.30	98.63
Shandong/Jining/543/2025	DQ473512	A30	89.99	97.53
Shandong/Jining/546/2025	FJ445111	A1	90.13	93.73
Shandong/Jining/933/2025	DQ473498	A10	89.58	97.92
Shandong/Jining/1056/2025	FJ445129	A40	89.63	97.90
Shandong/Jining/1090/2025	FJ445111	A1	90.24	93.73
Shandong/Jining/1544/2025	FJ445129	A40	90.09	97.55
Shandong/Jining/1547/2025	FJ445168	B91	89.30	96.62
Shandong/Jining/1550/2025	EF186077	C3	92.61	96.73
Shandong/Jining/1557/2025	FJ445129	A40	90.09	97.90
Shandong/Jining/1617/2025	EF186077	C3	92.61	96.73
Shandong/Jining/1619/2025	FJ445116	A13	91.03	95.86
Shandong/Jining/1621/2025	EF186077	C3	92.61	96.73
Shandong/Jining/1630/2025	DQ473496	A49	88.93	95.41
Shandong/Jining/1694/2025	X02316	A2	87.75	92.93
Shandong/Jining/1697/2025	JN614995	A105	95.37	99.65
Shandong/Jining/2048/2025	FJ445131	A43	89.76	96.27

### 3.6 Amino acid variation analysis

Analysis of amino acid variations in the VP1 region of the 30 rhinovirus strains (Supplementary Table 3) revealed that, compared with their respective reference strains, the number of amino acid substitution sites per strain ranged from 1 to 24, with a mean of 11 and a median of 9. Among the 22 RV-A strains, the number of amino acid substitution sites ranged from 1 to 24. Strain Shandong/Jining/1697/2025 (genotype A105) exhibited the fewest substitutions, with only one amino acid change (N90D), whereas strains Shandong/Jining/2310/2024 and Shandong/Jining/2362/2024 (both genotype A31) exhibited the most, each with 24 amino acid substitutions. The 3 RV-B strains had 10–19 amino acid substitutions, and the 5 RV-C strains had 4–13 amino acid substitutions.

With respect to the distribution of substitution sites, the majority of amino acid substitutions were concentrated in surface-exposed regions of the VP1 protein, involving sites associated with antigenic epitopes (Cordey et al., 2010; Palmenberg et al., 2009). Several strains exhibited substitutions at known functional sites: for example, strain Shandong/Jining/2416/2024 (genotype C13) had an I→A substitution at position 136, and strain Shandong/Jining/1694/2025 (genotype A2) had an N→K substitution at position 204. Overall, the rhinoviruses circulating in this region exhibited varying degrees of amino acid variation in the VP1 region, reflecting ongoing viral evolution. Nevertheless, the amino acid similarity between most study strains and their respective reference strains remained high (>91%).

## 4 Discussion

In this study, 2,724 ILI specimens collected in Jining City during 2024–2025 were screened for ten respiratory viruses. Influenza virus had the highest detection rate (14.98%), followed by SARS-CoV-2 (7.05%), with rhinovirus ranking third (3.71%). This respiratory virus spectrum is broadly consistent with contemporaneous reports from other regions of China (Jiang et al., 2024; Li et al., 2021, 2023; Yang et al., 2026a; Yin et al., 2024). Notably, the RV-positive rate in 2025 (5.53%) was significantly higher than that in 2024 (1.95%; *P* < 0.001), a phenomenon consistent with the overall trend of enhanced respiratory virus activity following the optimization and relaxation of COVID-19 prevention and control measures (Dou et al., 2025; Wu et al., 2026). NPIs implemented during the COVID-19 pandemic effectively suppressed the transmission of multiple respiratory viruses, including rhinovirus. Following the lifting of NPI measures, increased population susceptibility led to a rebound in viral activity, and multiple studies have reported similar “compensatory epidemics” (Kuitunen et al., 2021). Seasonal distribution analysis revealed that RV was detected sporadically throughout 2024, with no statistically significant inter-seasonal differences, whereas 2025 exhibited marked seasonality, with an autumn positivity rate of 11.38% and a November single-month positivity rate reaching 23.53%, forming a conspicuous epidemic peak. This transition from a sporadic to a concentrated outbreak pattern may be attributable to the following factors. First, the cumulative effect of the population immunity gap following the relaxation of NPI measures, particularly the decline in population-level immunity against predominant species such as RV-A. Second, climatic conditions in autumn 2025 in this region may have favored rhinovirus transmission – studies have shown that decreased relative humidity and lower temperatures can enhance the stability of rhinovirus in aerosols (Pica and Bouvier, 2012). Third, genetic variation within the virus may influence its transmission fitness to some extent. RV-C has been increasingly reported in recent years to be associated with more severe lower respiratory tract symptoms (Bochkov et al., 2011; Miller et al., 2009). Notably, the wider phylogenetic distribution of RV-C strains in 2025 compared to those in 2024 suggests a potential increase in the genetic diversity of RV-C strains; however, its association with the epidemic peak in the autumn of 2025 remains unclear and warrants further validation through longitudinal monitoring with larger sample sizes and functional assays (Benschop et al., 2021).

Molecular typing results demonstrated that the 30 sequenced strains belonged to RV-A (73.33%), RV-B (10.00%), and RV-C (16.67%), with RV-A as the overwhelmingly predominant species, consistent with the majority of domestic and international reports (Cui et al., 2024). RV-A in this study encompassed 17 genotypes and RV-C encompassed 3 genotypes. Such high genotypic diversity suggests the potential co-circulation of multiple transmission chains in this region (Cui et al., 2024; Goya et al., 2025), which may be related to Jining’s role as a transportation hub with substantial population mobility (Yang et al., 2026b; Zhao et al., 2025). Furthermore, among the RV-A genotypes, A40 (3 strains), A1, A30 and A31 (2 strains each) were the predominant circulating genotypes, yet the phylogenetic distances among genotypes were considerable, with no clearly dominant clonal group – a pattern consistent with the typical multi-genotype co-circulation model of respiratory viruses (Berginc et al., 2024; Robertson et al., 2021).

Phylogenetic analysis is an essential tool for elucidating viral evolutionary patterns and transmission dynamics. In this study, ML phylogenetic trees were constructed based on both whole-genome and VP1 region sequences, and the results from both analyses were highly concordant, showing that the three rhinovirus species form independent evolutionary clades. The distribution patterns of 2024 and 2025 strains within the phylogenetic trees indicate that the 2025 strains were distributed across a markedly broader range, with some strains forming independent evolutionary subclades, suggesting the accumulation of substantial genetic variation during continued circulation (Ratnamohan et al., 2016). This finding corroborates the epidemiological observation of a significantly elevated RV detection rate in 2025, providing molecular-level evidence for understanding the evolution–epidemic association of rhinovirus in this region.

Genetic distance and similarity analyses further quantified the degree of variation among the study strains. The VP1 region nucleotide similarity between the 30 strains and their respective reference strains ranged from 86.08% to 95.37% (mean, 89.93%), and amino acid similarity from 91.58% to 99.65% (mean, 96.13%). Amino acid similarity was consistently higher than nucleotide similarity, indicating that the majority of nucleotide substitutions in the VP1 region are synonymous, reflecting the functional constraint on the VP1 protein under natural selection pressure (Kistler and Bedford, 2021). Notably, individual strains exhibited distinctive variation patterns. For example, strain Shandong/Jining/1697/2025 (genotype A105) differed from its reference strain by only one amino acid substitution (N90D), with nucleotide and amino acid similarities as high as 95.37% and 99.65%, respectively, suggesting that this strain may represent a recently emerged circulating variant. In contrast, strains Shandong/Jining/2310/2024 and Shandong/Jining/2362/2024 (both genotype A31) each harbored 24 amino acid substitutions, representing a relatively high degree of variation. This marked intra-genotype variation in substitution burden reflects the heterogeneity of evolutionary rates within the rhinovirus population (Aris-Brosou and Yang, 2002).

Analysis of amino acid substitution sites revealed that the majority of substitutions were concentrated in surface-exposed regions of the VP1 protein, involving sites associated with known antigenic epitopes. For instance, strain Shandong/Jining/2416/2024 (genotype C13) exhibited an I→A substitution at position 136, and strain Shandong/Jining/1694/2025 (genotype A2) exhibited an N→K substitution at position 204. These sites are located within the “canyon” region of the VP1 protein, which is intimately involved in binding to viral receptors such as ICAM-1 or LDLR (Basnet et al., 2019). However, the amino acid similarity between the majority of strains and the reference strains remained at a relatively high level of >91%, suggesting that currently circulating rhinoviruses may be antigenically conserved. Cross-immunity established by previous infections may still confer a certain degree of protection, although this hypothesis requires further validation through serological or neutralization assays (Glanville and Johnston, 2015). Given the high number of rhinovirus genotypes and the lack of cross-protection among types, continuous molecular surveillance is essential for the timely detection of emerging variant strains that may possess immune escape capabilities.

Compared with recent studies from other provinces and cities in China, the species composition and genotype diversity observed in this study demonstrate favorable comparability. During the acute respiratory tract infection (ARTI) surveillance conducted across 35 sentinel hospitals in Beijing from 2018 to 2022, the detection rate of rhinoviruses (RVs) was 6.5%. Among the genotyped strains, RV-A, RV-B, and RV-C accounted for 68.8%, 11.7%, and 19.5% respectively, with a total of 105 genotypes detected and no single dominant genotype (Wang et al., 2024). This profile – characterized by “RV-A dominance, a lower proportion of RV-B, and concurrent transmission of multiple genotypes” – is consistent with the findings from Jining. Similarly, an acute respiratory infection (ARI) study in Shanghai from 2012 to 2020 reported that RV-A, RV-B, and RV-C accounted for 68.13%, 20.00%, and 11.88% respectively (Jiang et al., 2022). A multi-center study on community-acquired pneumonia (CAP) in Chinese children also showed a predominance of RV-A (109/209) followed by RV-C (80/209), alongside amino acid substitutions identified within potential neutralizing epitopes of the VP1 protein in certain strains (Ai et al., 2022). Long-term pediatric hospitalization surveillance in Suzhou and inpatient ARI research in Shenzhen from 2019 to 2024 both demonstrated enhanced RV activity following the adjustment of non-pharmaceutical interventions (NPIs), accompanied by distinct spring-autumn dual peaks or an autumn-dominated peak (Liu et al., 2025; Shi et al., 2025), which aligns closely with the peak observed in Jining in the autumn of 2025, particularly in November.

Compared with relevant studies from neighboring countries, factors such as regional climate and study populations can significantly influence detection rates and the proportion of viral species or types. In ARI surveillance conducted in Vietnam from 2012 to 2016, the RV detection rate was 13%, suggesting that neighboring nations may serve as important sources of introduction for diverse respiratory viral lineages (Lu et al., 2020). In Mongolian surveillance from 2008 to 2013, the RV detection rate was 11.0%, with comparable proportions of RV-A and RV-C (47.1% and 44.7%, respectively) and RV-B accounting for only 8.2% (Tsatsral et al., 2015). Compared with these findings, Jining exhibited a higher proportion of RV-A (73.33%) and a lower proportion of RV-C (16.67%). However, such discrepancies may be attributed to variations in climate, pediatric versus general population composition, case definitions, and genotyping strategies (targeting VP1, VP4/VP2, or the 5’ UTR).

Several limitations of this study should be acknowledged. First, our data were derived from a sentinel surveillance system for influenza-like illness, which only recorded basic demographic information and collection dates of the cases, lacking detailed evaluations of clinical symptoms, disease severity, and co-infections. This limitation precludes an in-depth analysis of the clinical implications associated with different rhinovirus genotypes. Future prospective clinical studies should be designed to systematically collect comprehensive clinical data to better elucidate the relationship between rhinovirus genotypic diversity and disease manifestations. Second, selecting rhinovirus-positive samples with low *Ct* values for whole-genome sequencing constituted a non-random sampling strategy, which inherently limits the generalizability of the genetic diversity findings. Such low Ct-based screening may skew the sequencing data toward samples with high viral loads, potentially underestimating the genetic diversity of strains with low viral loads in this study. Third, although whole-genome sequencing data were successfully obtained, our analysis predominantly focused on the VP1 region, failing to fully exploit evolutionary information at the whole-genome level, such as the variation characteristics of non-structural protein-coding regions and their potential impacts on viral replication and pathogenicity. A more comprehensive and systematic mining of the whole-genome data will be a primary focus of our subsequent research. Fourth, the statistical analyses in this study were primarily descriptive, utilizing only chi-square tests for inter-group comparisons, without employing more sophisticated statistical models, such as multivariate regression or time-series analyses, to assess the associations between rhinovirus genotypes and factors such as seasonality and demographics. Therefore, while the current statistical strategy reflects the fundamental trends of rhinovirus circulation, it remains insufficient to systematically reveal the interactions among various factors and their underlying determinants. Future studies should incorporate more refined statistical methodologies to comprehensively elucidate the driving forces and transmission dynamics of rhinovirus epidemics.

In conclusion, RV-A was the predominant rhinovirus species circulating in Jining City during 2024–2025, with high genotypic diversity. The RV detection rate in 2025 increased significantly compared with 2024, accompanied by a pronounced autumn epidemic peak. Whole-genome and VP1 region phylogenetic analyses revealed the rich genetic diversity and ongoing evolutionary dynamics of rhinoviruses in this region. This study provides the systematic report of the molecular epidemiological characteristics of rhinovirus in Jining City, contributing important baseline data for the formulation and refinement of regional respiratory virus prevention and control strategies. We recommend the continued strengthening of molecular surveillance for rhinovirus to dynamically track epidemiological and evolutionary trends, thereby providing scientific support for vaccine development and the optimization of clinical diagnostic and therapeutic strategies.

## Data Availability

The datasets presented in this study can be found in online repositories. The names of the repository/repositories and accession number(s) can be found in the article/Supplementary material.

## Supplementary material

Supplementary Table 1 Seasonal, temporal, and sex distribution of RV-positive cases among ILI cases in Jining City, 2024–2025. RV Pos: rhinovirus-positive cases; RV Neg: rhinovirus-negative cases.

Supplementary Table 2 Amino acid variations in the VP1 region of rhinovirus strains in Jining City, 2024–2025.

Supplementary Table 3 Whole-Genome Recombination Analysis. To screen for recombination events across the whole-genome sequences of the RV-A, RV-B, and RV-C groups (comprising both study samples and reference sequences), RDP4 software was employed. Detection was performed using six algorithms built into the software: RDP, GENECONV, BOOTSCAN, MaxChi, Chimaera, and SisScan. To enhance the reliability of the findings, a recombination event was considered positive only when it was simultaneously supported by at least three algorithms (P < 0.05, with Bonferroni correction), and all detected events were manually verified on a case-by-case basis.

Supplementary Table 4 GenBank accession numbers.
